# Adenoma malignum of the uterine cervix: report of four cases

**DOI:** 10.1186/1477-7819-11-168

**Published:** 2013-07-26

**Authors:** Eun Young Ki, Seung Won Byun, Jong Sup Park, Sung Jong Lee, Soo Young Hur

**Affiliations:** 1 Department of Obstetrics and Gynecology, The Catholic University of Korea, Seoul St Mary’s Hospital, Banpo-dong, Seocho-gu 137-140, Seoul, Korea; 2 Department of Obstetrics and Gynecology, The Catholic University of Korea, St Vincent Hospital, Paldal-gu, Suwon city, Korea

**Keywords:** Adenoma malignum, Minimal-deviation adenocarcinoma, Uterine cervix

## Abstract

Adenoma malignum (AM) of the cervix is a rare disease and it is difficult to diagnose due to the deceptively benign appearance of the tumor cells. These lesions have mucin-rich cystic lesions and are usually situated deep in the cervix. Since AM is very rare, standard screening tests, diagnostic tools and treatments have not yet been established. Radiologically, it mimics multiple nabothian cysts as a benign-looking tumor. Histologically, AM is a well-differentiated adenocarcinoma and could be misdiagnosed as a benign lesion. These findings make a preoperative diagnosis of AM difficult and can result in surgery being performed based on a misdiagnosis. We report here on four cases of pathologically confirmed AM.

## Background

Adenoma malignum (AM), also called minimal-deviation adenocarcinoma, has been reported in the literature since Gusserow first described a very highly differentiated adenocarcinoma of the uterine cervix in 1870 [1,2]. The word ‘adenoma’ describes the histologic appearance and the word ‘malignum’ the virulent and fatal progression of this tumor. It is a subtype of mucinous adenocarcinoma of the cervix and its prevalence is very low (1% to 3% of all cervical adenocarcinomas) [3,4]. Because of its rarity, many authors have reported on its treatment and prognosis, but the standard treatment has not yet been established. AM exhibits an endophytic rather than exophytic growth pattern. In addition, it mimics multiple nabothian cysts as a benign-looking tumor [5-7]. These findings make a preoperative diagnosis of AM difficult and can result in surgery being performed based on a misdiagnosis. We report here on four cases of pathologically confirmed AM.

## Case presentation

### Case 1

**Figure 1:**
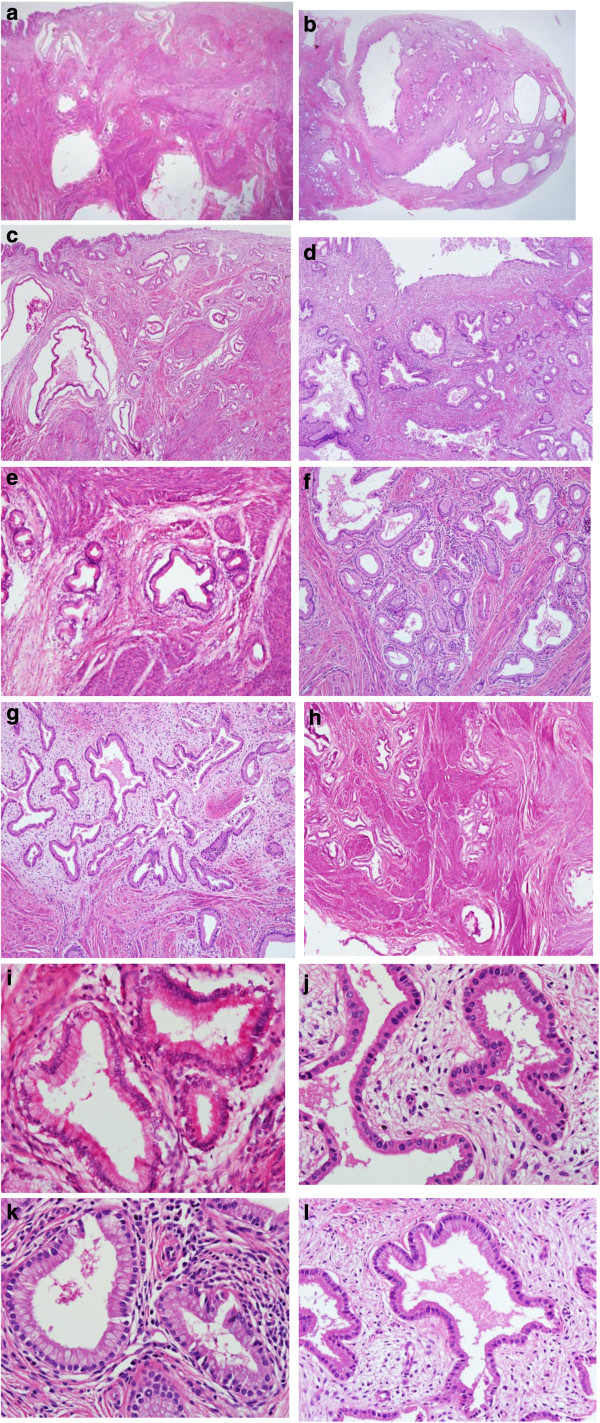
**Photomicrographs of large cysts showing the columnar epithelial lining. (a**–**d)** Numerous irregularly shaped mucinous glands are seen, which are infiltrating below the surface of the endocervix (H&E stain, ×40, cases 1 to 4). **(e**–**h)** Diffusely infiltrating mucinous glands and well-formed glands are arranged irregularly, showing heterogeneity in size and shape. The glands show mild desmoplastic stromal reactions associated with lymphocytic infiltration (H&E stain, ×100, cases 1 to 4). **(i**–**l)** The differentiation of the glands is as good as that of normal endocervical glands (H&E stain, ×400, cases 1 to 4).

A 54-year-old woman presented at our hospital with several months of leukorrhea and menorrhagia. Physical examination revealed normal external genitalia and a large amount of clear mucoid fluid in the posterior vaginal fornix. Her cervix was grossly normal in appearance except for the profuse mucoid discharge. Her uterus was normal in size, shape and consistency. No adnexal masses were palpable. Transvaginal sonography showed several small intramural myomas and nabothian cysts. The patient underwent a total abdominal hysterectomy and bilateral adnexectomy. Pathologic examination of the specimen revealed AM of the cervix (Figure 1a, e and i) and small intramural myomas. Examination of the ovaries revealed a fibroma and a follicular cyst. The patient had an uneventful postoperative course and did not receive adjuvant therapy. She is still alive 14 years after surgery.

### Case 2

**Figure 2:**
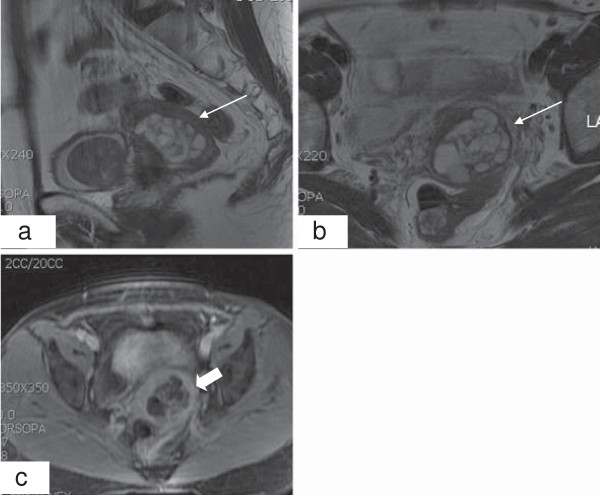
**Magnetic resonance imaging for case 2. (a**,**b)** T2-weighted sagittal and axial images show multiple small cystic lesions with high signal intensity in the enlarged cervical stroma (narrow arrows). **(c)** A gadolinium-enhanced T1-weighted axial image shows multiple cystic lesions of unequal size. The abnormally enhanced area is the septum of the tumor (thick arrow).

A 53-year-old woman presented at our hospital with vaginal bleeding for several weeks along with low abdominal pain and fever for one day. She had a subtotal hysterectomy one year prior because of a huge myoma. Physical examination revealed no abnormal findings. The cervix was grossly normal. Magnetic resonance imaging (MRI) revealed multiple cystic lesions of the cervix that were of high T2 and a smooth marginated mass around the bladder that was of low T2 isosignal intensity (Figure 2). The patient underwent stump resection and bladder mass excision. Pathologic examination of the specimen revealed AM of the cervix (Figure 1b, f and j) along with an endocervical polyp and a leiomyoma. Examination of the ovaries revealed unremarkable findings. The patient ran an uneventful postoperative course, did not receive adjuvant therapy and is still alive six years after surgery.

### Case 3

A 39-year-old woman presented at our hospital with profuse vaginal discharge. Physical examination revealed normal external genitalia. Her cervix was grossly normal in appearance except for the profuse mucoid discharge. Her uterus was normal in size, shape and consistency. In order to evaluate the source of this discharge, the patient underwent colposcopy. The biopsy result was consistent with cervical intraepithelial neoplasia I. Transvaginal sonography did not reveal any abnormal findings. She underwent conization and pathologic examination of the conization specimen revealed multiple well-differentiated but irregularly shaped glands with invasion of the resection margin (Figure 1c, g and k). These findings were consistent with AM and the patient underwent a type I hysterectomy with pelvic lymph node sampling. Further examination of the cervix from the hysterectomy specimen showed no abnormal findings. The patient had an uneventful postoperative course and did not receive adjuvant therapy. She is still alive two years and nine months after surgery and has had no recurrences.

### Case 4

A 42-year-old woman who suffered profuse watery vaginal discharge for three years and amenorrhea for six months visited our hospital. Her cervix was grossly normal in appearance except for the profuse mucoid discharge. Her uterus was slightly enlarged. Computed tomography showed a mass-like enlargement of the uterine cervix with multiple internal cystic foci. She underwent a total laparoscopic hysterectomy. Grossly, there were several subserosal myomas in the fundus and multiple small nabothian cysts in the cervix. Pathologic examination of the specimen revealed AM of the cervix (Figure 1d, h and l). Additionally, the patient underwent laparoscopic pelvic lymph node and para-aortic lymph node dissection. Pathologic examination of the specimen revealed no abnormal findings. The patient had an uneventful postoperative course and is still alive five months after surgery without any recurrences.

### Discussion

After the first description of AM of the uterine cervix by Gusserow, McKelvey and Goodlin reported five AM cases [8]. Recently, a few cases of complicated AM have been reported in the literature.

The major clinical symptoms of AM are profuse mucoid vaginal discharge and irregular/contact vaginal bleeding [5,7]. Hirai *et al*. [10] reported profuse vaginal discharge in all cases, Gilks *et al*. [9] reported it in 11 of 26 cases and Lim *et al*. [5] reported it in 6 of 18 cases. In our four cases, three had profuse watery or mucoid vaginal discharge and two had irregular vaginal bleeding. When there are grossly normal vaginal and cervical findings in such patients, the presence of multilocular cystic masses in the uterine cervix, which can be detected by transvaginal sonography, may help to make a clinical diagnosis of AM [5,7]. The echogenicity of solid lesions tends to be heterogenous unlike leiomyomas. Park *et al*. [11] reported that ultrasound imaging with Doppler examination is more efficient and accurate in the evaluation of multilocular lesions of the uterine cervix, and in their study Doppler examination revealed that AM had an increased intralesional vascularity. Recently, magnetic resonance imaging has been shown to be useful for the diagnosis of AM. This imaging reveals multiple irregular cystic lesions, which mimic benign nabothian cysts. However, magnetic resonance imaging of AM characteristically presents with medium-to-high signal intensity on T1WI images and markedly high signal intensity on T2WI images [3,6,12].

A preoperative histologic diagnosis of AM is often difficult because of the characteristic of AM. The screening test for cervical cancer is cytologic evaluation by a Papanicolaou test [13]. Because the lesion is located deep in the endocervix and exhibits an endophytic growth pattern, it is difficult to make an accurate cytological diagnosis [4]. Ishii *et al*. [4] and Chang *et al*. [7] reported similar cytologic features of AM: (1) the frequent appearance of large sheets of cells with a honeycomb pattern and a palisading arrangement at the periphery, (2) abnormal glandular cells with abundant cytoplasm showing atypia and benign-appearing glandular cells, and (3) slightly enlarged mucous cells with ovoid nuclei. Histologic examination reveals well-spaced, deeply invasive branching glands lined with uniform columnar mucin-distended cells and infrequent foci of less well-differentiated neoplastic cells [14]. Kaminski *et al*. [15] reported three types of AM, having endometrioid, mucinous or clear cell types. Unlike adenocarcinoma, the histologic features of AM rarely have mitotic figures, pseudo-stratification or atypia. There have been some reports of mucinous gland proliferation of varying sizes, shapes and densities, which often extends deeper than normal into cervical tissue [7,14]. Intraluminal papillary projections of the epithelium and an edematous or desmoplastic stromal response are occasionally presented [16]. Because of the well-differentiated appearance, diagnosis is often difficult to make. It is important to differentiate AM from other lesions, such as normal endocervix, microglandular hyperplasia, adenocarcinoma *in situ* and well-differentiated endocervical adenocarcinoma [7,16]. Immunohistochemical studies have been used to make more accurate diagnoses. Ishii *et al*. [4] reported that mucinous AM cells are positive for HIK 1083. Urusagi *et al*. [17] reported that 90% of AM patients are positive for this antibody. Mercer *et al*. [16] showed that by using the carcinoembryonic antigen (CEA), AM can be distinguished from well-differentiated adenocarcinoma and other lesions [16].

In recent reports, the treatment of AM has been similar to that of endocervical adenocarcinomas and surgery is the standard treatment modality for both [5,7,18,19]. Types of surgery vary from author to author. In a report by Lim *et al*. [5], five patients underwent type I hysterectomy without pelvic lymph node dissection (PLND), five patients underwent type I hysterectomy with PLND, and eight patients underwent type III radical hysterectomy (RH) with PLND. Ryuichi *et al*. [18] reported four cases of AM: one underwent PLND and external radiation, one underwent radical hysterectomy and external radiation, one underwent radical hysterectomy omentectomy and combination chemotherapy and the final patient underwent radical hysterectomy, external radiation and combination chemotherapy. In our cases, two underwent simple hysterectomy and bilateral adnexectomy (BA), one underwent type I hysterectomy and pelvic node sampling, and one patient underwent simple hysterectomy and PLND (Table 1).

**Table 1:** Initial treatment and survival

	**Type of surgery**	**Adjuvant treatment**	**Survival**
Lim *et al*. (18 cases) [5]	Type I RH (5/18)	None	
	Type I RH + PLND (5/18)	None	Mean follow-up period: 49.2 months
	Type III RH + PLND (8/18)	CCRT (4/8)	Progression: 2/18 Death: 2/18
		ERT (3/8)	
		CTx (1/8)	
Ryuichi *et al*. (4 cases) [18]	RH	ERT + CTx	5 years
		ERT + CTx	
	RH + omentectomy	ERT	6 months
	RH		36 months
	Lymph node dissection		15 months
Bulmer *et al.* (2 cases) [19]	RH + PLND	None	3 years
	Vaginal TH	ERT	7 years
Gotoh *et al*. (1 case) [3]	TH	None	2 years (NED)
Koo *et al*. (1 case) [20]	RH	CTx	12 months (NED)
Chang *et al*. (5 cases) [7]	None	ERT	6 months (DOD)
	RH	ERT + CTx	46 month (DOD)
	TH	ERT + CTx	18 months (DOD)
	RH	ERT	5 years (NED)
	RH	ERT + CTx	3 years (NED)
Ki *et al*. (4 cases)	TH + BA	None	14 years (NED)
	TH + BA	None	6 years (NED)
	Type I RH + pelvic lymph node sampling	None	33 months (NED)
	TH + PLND	None	5 months (NED)

*BA*: bilateral adnexectomy, *CCRT*: concurrent chemoradiation therapy, *CTx*: chemotherapy, *DOD*: died of the disease, *ERT*: external radiation, *NED*: no evidence of disease, *PLND*: pelvic lymph node dissection, *RH*: radical hysterectomy, *TH*: total hysterectomy.

A previous report showed that AM cases have unfavorable prognoses. Hirai *et al*. [10] indicated that since most AM cases are detected at advanced stages and are often treated on the basis of clinical diagnoses their prognoses are poor.

## Conclusions

Since AM is very rare, standard screening tests and diagnostic tools have not yet been established. Histologically, AM is a well-differentiated adenocarcinoma and could be misdiagnosed as benign lesions. We experienced four favorable cases, which we have reported with a brief review of the literature.

## Consent

Written informed consent was obtained from each patient for publication of this case report. The study was approved by the Institutional Review Board (KC12ZISE0770).

## Abbreviations

AM: adenoma malignum; BA: bilateral adnexectomy; CCRT: concurrent chemoradiation therapy; CTx: chemotherapy; DOD: died of the disease; ERT: external radiation; NED: no evidence of disease; PLND: pelvic lymph node dissection; RH: radical hysterectomy; TH: total hysterectomy.

## Competing interests

The authors declare that they have no competing interests.

## Authors’ contributions

EYK wrote the initial draft. SYH, JSP and SJL performed the surgery and helped collect clinical information. SYH and SWB designed the study and helped write the manuscript. All authors read and approved the final manuscript.
